# Delayed post-hypoxic leukoencephalopathy following alcohol consumption and cardiopulmonary arrest

**DOI:** 10.1016/j.radcr.2021.07.035

**Published:** 2021-08-09

**Authors:** I. Tahir, M.U. Islam

**Affiliations:** a University College Cork School of Medicine, Cork, Ireland, College Rd Cork Ireland; b Department of Radiology, Fraser Health, Abbotsford, British Columbia

**Keywords:** Delayed post hypoxic leucoencephalopathy, Neuroradiology, Hypoxic brain injury

## Abstract

Delayed post hypoxic leukoencephalopathy (DPHL) is a rare consequence of hypoxic brain injury that occurs several days to weeks following an initial hypoxic insult. Most of the previously published cases occur in the setting of drug overdoses or carbon monoxide poisoning, where the incidence of DPHL is as high as 3%. Our case depicts a patient with delayed hypoxic brain injury following cardiac arrest with cardiopulmonary resuscitation. Initial neuroimaging was normal, and a repeat MRI scan six days later revealed DWI changes consistent with DPHL. Our patient remained comatose throughout his clinical course until his eventual death nine days after the initial incident. The autopsy confirmed hypoxic-ischemic brain injury with co-existent Wernicke's encephalopathy, a known consequence of alcohol use disorder. This case outlines the clinical course of DPHL accompanied by the unique neuroimaging features that distinguish it from conventional hypoxic-ischemic brain injury.

## Introduction

Global hypoxic brain injury occurs due to inadequate cerebral oxygen perfusion and can have devastating consequences, including permanent neurologic dysfunction, coma, or death [1]. The patterns of brain injury following hypoxia vary depending on the underlying insult. Generally, acute anoxic brain injury is seen following hypoxia due to asphyxiation, hypoventilation, cardiopulmonary arrest, profound hypotension, and carbon monoxide (CO) poisoning [2]. In such cases, the injury occurs by cytotoxic edema that predominantly involves grey matter [3]. In cases of delayed hypoxic leukoencephalopathy (DPHL), the pattern of injury involves the white matter and usually occurs weeks following an initial hypoxic insult [1]. Although the mechanism for white matter involvement in DPHL is not understood, several theories exist including direct myelinotoxity [4], disruptions in cellular respiration [4], arylsulfatase A deficiency [2], and damage from reactive oxygen species [5]. Classic symptoms include delirium, personality changes, akinetic mutism, and parkinsonism [4]. An interim improvement in clinical symptoms is frequently observed, while seizures are a rare consequence [1].

## Case report

A 43-year-old male with no significant past medical history consumed several units of alcohol one evening and had left the company of others for 30 minutes when he was found collapsed and unconscious on the ground. CPR was performed by Emergency Medical Services. He was also intubated at the scene due to decreased respiratory effort and a low Glasgow Coma Scale (GCS).

In the emergency department, he remained intubated, mechanically ventilated, and unresponsive. He was hemodynamically stable. An initial computed tomography (CT) scan failed to document any acute intracranial abnormality to explain his coma. An echocardiogram was unremarkable. Lumbar puncture (LP), electroencephalography (EEG), and magnetic resonance imaging (MRI) were performed. LP revealed normal cell counts with no xanthochromia. EEG following admission revealed paroxysmal epileptiform activity, and repeat studies continued to document ongoing epileptiform activity despite no clear status epilepticus. He was commenced on multiple anticonvulsants.

**Fig. 1:**
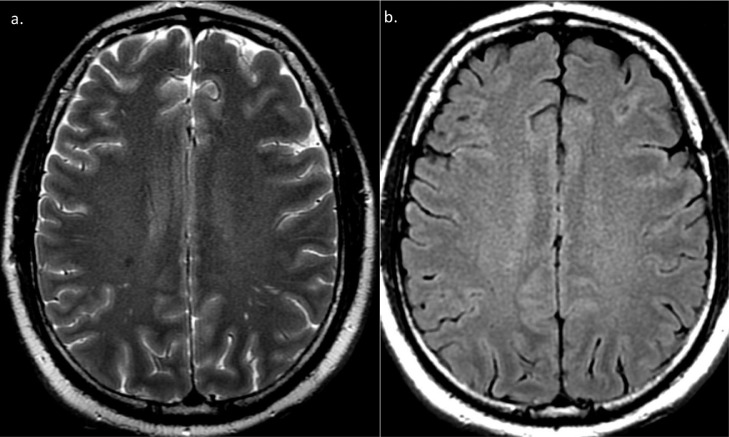
T2 (a) and FLAIR (b) MRI sequences at the level of centrum semiovale showing normal appearance on initial MRI.

**Fig. 2:**
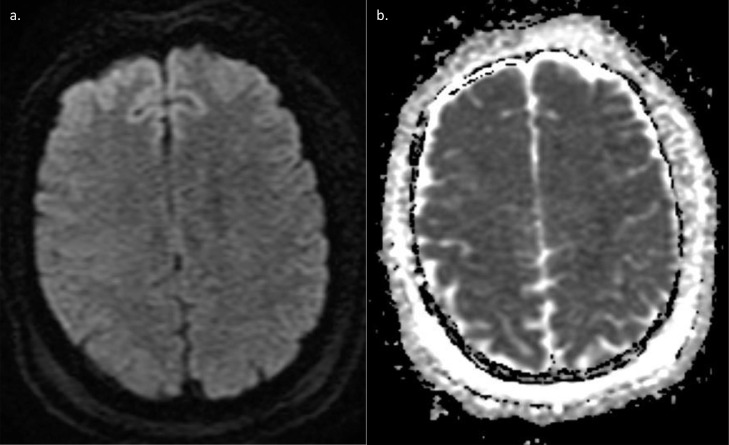
(a) DWI and (b) ADC on initial MRI examination. No evidence of restricted diffusion is seen.

MRI of the head on day 1 of admission was normal (Fig. 1, Fig. 2), and no structural cause of epileptic activity was identified. Laboratory investigations including a complete blood count, electrolytes, renal function, and C-reactive protein were non-contributory. His sodium was 144mmol/l, lactate 1.7 mmol/l, and pH 7.35.  He had mildly deranged liver function tests (ALT 153 IU/L, AST 122 IU/L, GGT 305 IU/L) in keeping with alcoholic liver disease. Viral hepatitis serology was negative. The patient had a raised troponin (0.31 mcg/ml) but no signs of acute myocardial infarction on his ECG. Serum toxicology screening showed a raised blood alcohol level (48.8mmol/l) and benzodiazepines but was negative for opiates and amphetamines.

**Fig. 3:**
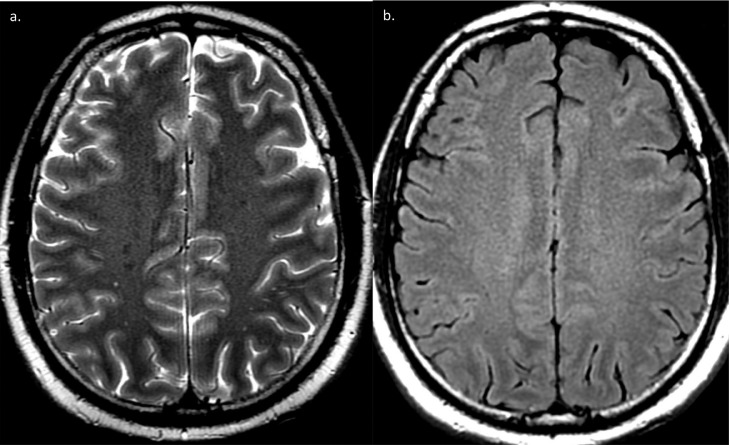
T2 (a) and Flair (b) MRI sequences at follow up MRI, six days after initial MRI showing no detectable signal changes.

**Fig. 4:**
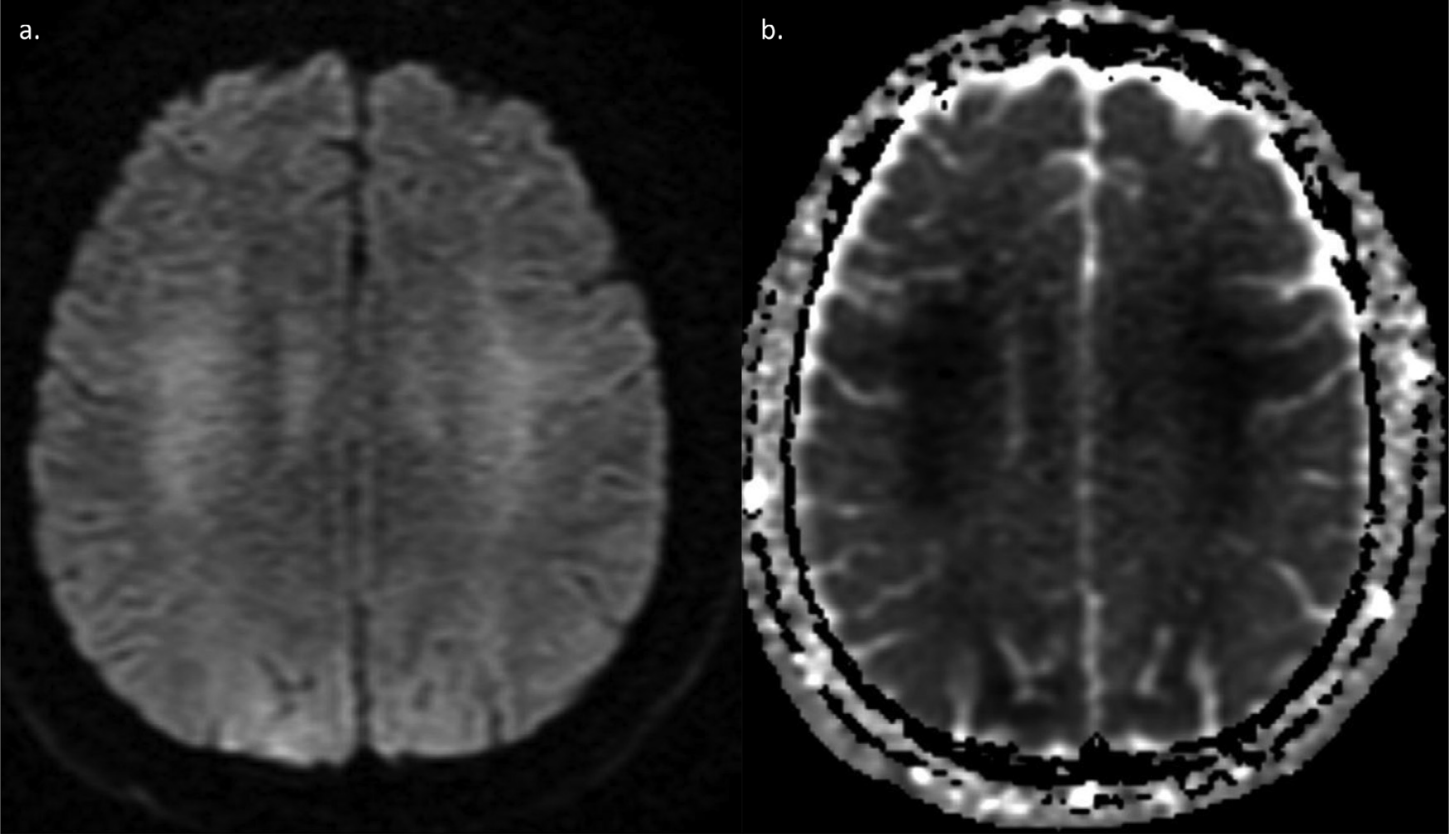
DW-MRI sequence (a) shows increased signal in bilateral centrum semi ovale and posterior parietal cortex and subcortical white matter. Corresponding ADC map (b) shows low signal in the same regions confirming diffusion restriction.

He remained clinically unresponsive and comatose for the subsequent days. Clinically, he was suspected to have an underlying anoxic brain injury given the delayed resuscitation efforts, although normal initial imaging was unusual. A repeat MRI performed six days following initial imaging **(**Fig. 3, Fig. 4**)** revealed increased diffusion weighted imaging (DWI) signal and corresponding low apparent diffusion coefficient (ADC) signal changes involving bilaterally symmetrical areas, predominantly the white matter of centrum semiovale and bilateral posterior parietal grey and white matter.

Differential diagnoses included acute anoxic brain injury, osmotic demyelination syndrome, posterior reversible encephalopathy syndrome, and DPHL. DPHL was the final diagnosis given to our patient since it was most consistent with the delayed onset MRI changes and white matter involvement. The fact that the white matter changes were only seen on DWI sequence suggests that the changes were picked up early and not yet apparent on standard T2 and FLAIR sequences.

Our patient was treated with supportive measures. After no improvement on day nine of admission and following a discussion with his family, a decision was made to take him off mechanical ventilation due to his persistent vegetative state. The autopsy confirmed histological features of hypoxic-ischemic brain injury. There were also features of chronic alcoholism including acute Wernicke's encephalopathy with organizing early infarction of both mammillary bodies and tectum, and cerebellar atrophy consistent with chronic ethanol abuse. Ethanol was detected in antemortem blood and hepatic steatosis was also noted. Although the cause of our patient's cardiac arrest was never determined, it was the preceding hypoxic insult resulting in DPHL.

## Discussion

Our case depicts a male with Wernicke's encephalopathy due to an alcohol use disorder who developed DPHL several days following a cardiopulmonary arrest. Initial CT and MRI were normal, and the diagnosis of DPHL was made following a repeat MRI six days later. This was confirmed on autopsy, where co-existent Wernicke's encephalopathy was diagnosed.

The sequence of neurochemical events following cerebral hypoxic injury includes compensatory anaerobic glycolysis, activation of calcium channels, membrane depolarization, and subsequent presynaptic glutamate release [3]. This leads to widespread excitotoxicity and free radical formation, causing tissue apoptosis or necrosis [3]. The grey matter is preferentially affected as this is where most glutamatergic receptors are located [1]. These changes are seen on CT as diffuse cortical hypo-attenuation, loss of grey-white differentiation, and diffuse edema. Our patient did not have the classical features of hypoxic brain injury– he instead developed DPHL, a pattern of injury that primarily affects the white matter.

DPHL is a rare phenomenon seen in 2.8% of cases of hypoxic-ischemic brain injury due to CO inhalation [6]; although the overall incidence is unknown it is likely underdiagnosed. Most cases are observed in the setting of either CO poisoning or heroin overdose, suggesting that these may be directly myelinotoxic [4]. The precise pathophysiology of DPHL remains unclear, although in animal models it is reproducible by injection with potassium cyanide [4]. Both cyanide and CO poisoning inhibit ATP based cellular respiration and bind to cytochrome c oxidase, suggesting that the myelinotoxicity of CO poisoning may be mediated by this mechanism. A deficiency in the lysozyme arylsulfatase A has been proposed as a potential cause of DPHL [2]. However normal arylsulfatase A levels have been observed in patients who develop the condition [4,7]. A therapeutic effect of antioxidant therapy was observed in a prior case report, suggesting that reactive oxygen species may also play a role in the pathophysiology [5].

MRI features in our patient included restricted diffusion involving the bilateral centrum semi ovale, posterior parietal cortex and subcortical white matter. Changes were not noted on T1, T2, or T2/FLAIR MRI sequences. Since DW-MRI is the most sensitive sequence for picking up these changes, we suspect that they had not yet progressed to involve other sequences. Meyer proposed that the reason for delayed white matter changes may be due to turnover rates of myelin proteins [8]. This fits the clinical picture of most reported cases but differs from our case where DW-MRI changes were observed six days following the initial hypoxic insult, rather than the mean time of 2-3 weeks [9].

Although hypoxic-ischemic brain injury was suspected in our patient, it remains unclear why there was no grey matter involvement. This may reflect the fact that there was a mild-to-moderate degree of hypoxia since severe hypoxia would more likely involve the grey matter [9]. Prior cases have reported an interim period of improvement of neurologic symptoms preceding an abrupt clinical deterioration and white matter involvement on MRI [8]. Our patient remained comatose with no evidence of clinical improvement and developed persistent epileptiform brain activity, a known but uncommon sequela of DPHL [1]. Our patient also had co-existent Wernicke's encephalopathy that was diagnosed on autopsy but wasn't evident on either MRI, which has a 53% sensitivity for picking up the condition [10]. Raised serum alcohol and benzodiazepine levels were observed on toxicology and could be involved in the progression to DPHL based on prior cases [9,^11^,12].

## Learning Points

● Global hypoxic brain injury occurs in response to prolonged brain hypoxia, etiologies include cardiac arrest, hypoventilation, severe hypotension, and toxic injury from carbon monoxide poisoning or heroin abuse.
● Hypoxic brain injury in adults usually presents as acute anoxic brain injury, involving the grey matter.
● Delayed post hypoxic leukoencephalopathy (DPHL) is a rare consequence of global hypoxia. Common causes include carbon monoxide and heroin overdose.
● DW-MRI is the most sensitive sequence for characterizing DPHL and presents with increased signal in white matter regions with corresponding low apparent diffusion coefficient mapping.

## Informed Consent

Our patient was deceased several years prior to the writing of this case report, we were unable to contact the patients next of kin. Personal details and imaging features have been anonymized to protect our patient's identity.
